# Supplementary material for: Macrophages take up VLDL-sized emulsion particles through caveolae-mediated endocytosis and excrete part of the internalized triglycerides as fatty acids
Source: PLoS Biol. 2022 Aug 26;20(8):e3001516. doi: 10.1371/journal.pbio.3001516 (PMC9455861; doi:10.1371/journal.pbio.3001516)
Supplement: S1 Raw Images — (PDF) [file pbio.3001516.s002.pdf]

Original blots

RAW blot Fig 2a

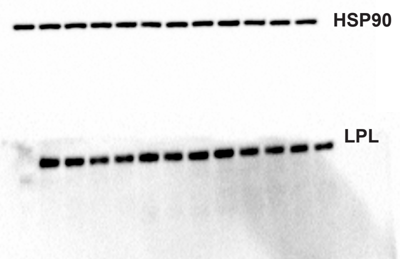

Loading order: CTRL/Heparin/VLDL-sized/
CHYL-sized/VLDL-sized+heparin/
CHYL-sized+heparin
(in biological duplicates)

RAW blot Fig 2d

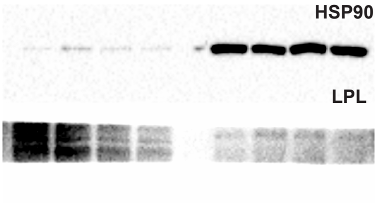

Loading order: siCTRL/si LPL (pellet)
siCTRL/siLPL (supernant)
(in biological duplicates)

RAW blot Fig 5a

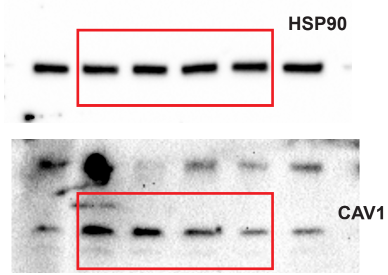

Loading order: siCTRL/si CAV1
(in biological duplicates)

RAW blot Fig 7d

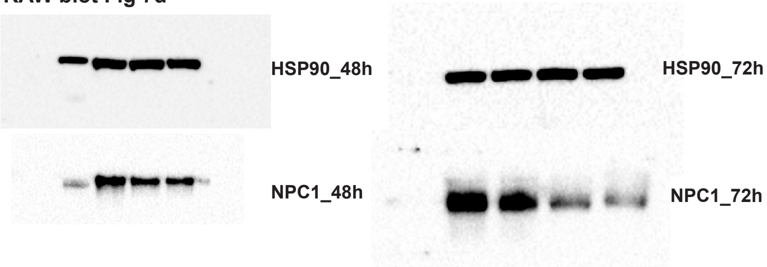

Loading order: siCTRL/si NPC1
(in biological duplicates)

RAW blot Fig 8b

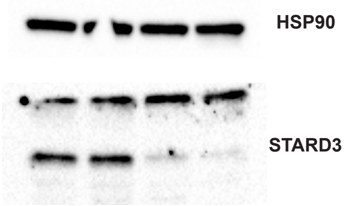

Loading order: siCTRL/si STARD3
(in biological duplicates)
