# Supplementary material for: Macrophages take up VLDL-sized emulsion particles through caveolae-mediated endocytosis and excrete part of the internalized triglycerides as fatty acids
Source: PLoS Biol. 2022 Aug 26;20(8):e3001516. doi: 10.1371/journal.pbio.3001516 (PMC9455861; doi:10.1371/journal.pbio.3001516)
Supplement: S1 Table — (DOCX) [file pbio.3001516.s004.docx]

**Table S1 Target sequences for siRNA used in the study**

| **siRNA** | **Contents** | **Target Sequence** |
| --- | --- | --- |
| ON-TARGETplus Human CAV1 (857) siRNA - SMARTpool | siRNA J-003467-06 | CUAAACACCUCAACGAUGA |
|  | siRNA J-003467-07 | GCAAAUACGUAGACUCGGA |
|  | siRNA J-003467-08 | GCAGUUGUACCAUGCAUUA |
|  | siRNA J-003467-09 | GCAUCAACUUGCAGAAAGA |
| ON-TARGETplus Human CAV2 (858) siRNA - SMARTpool | siRNA J-010958-05 | AGAUUGGGAUACUGUAAUA |
|  | siRNA J-010958-06 | GUAAAGACCUGCCUAAUGG |
|  | siRNA J-010958-07 | GUAGGACGAUGCUUCUCUU |
|  | siRNA J-010958-08 | UAUCAUUGCUCCAUUGUGU |
| ON-TARGETplus Human STARD3 (10948) siRNA - SMARTpool | siRNA J-017665-05 | GCGCAGGGACCGAUACUUG |
|  | siRNA J-017665-06 | GGCAAGACGUUUAUCCUGA |
|  | siRNA J-017665-07 | CAAGGGACUUCGUGAAUGU |
|  | siRNA J-017665-08 | GGAUGGUGCUGUGGAACAA |
| ON-TARGETplus Human NPC1 (4864) siRNA - SMARTpool | siRNA J-008047-05 | GGACAACUAUACCCGAAUA |
|  | siRNA J-008047-06 | GAAGAAAGCCCGACUUAUA |
|  | siRNA J-008047-07 | GCGAACGGCUUCUAAAUUU |
|  | siRNA J-008047-08 | GAUGAGACCAAUUGUGAUA |
| ON-TARGETplus Human LPL (4023) siRNA - SMARTpool | siRNA J-008970-05 | GCAGGAAGUCUGACCAAUA |
|  | siRNA J-008970-06 | CAUGACAAGUCUCUGAAUA |
|  | siRNA J-008970-07 | CCUACAAAGUCUUCCAUUA |
|  | siRNA J-008970-08 | GGGCUCUGCUUGAGUUGUA |
