# Supplementary figures and images for: Macrophages take up VLDL-sized emulsion particles through caveolae-mediated endocytosis and excrete part of the internalized triglycerides as fatty acids
Source: PLoS Biol. 2022 Aug 26;20(8):e3001516. doi: 10.1371/journal.pbio.3001516 (PMC9455861; doi:10.1371/journal.pbio.3001516)

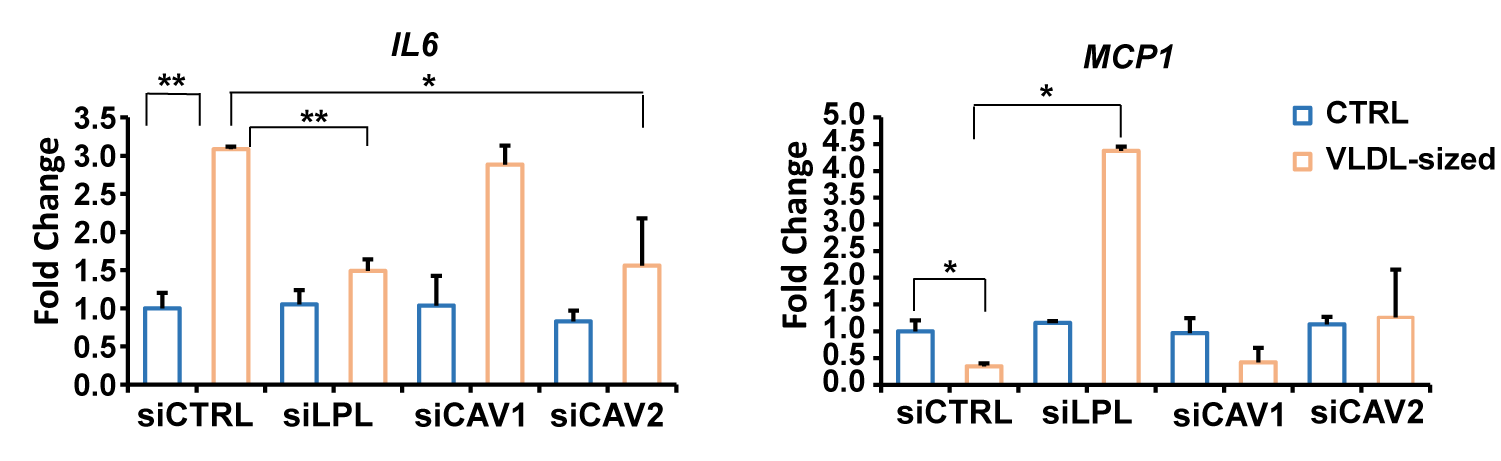

Supplement: S1 Fig — Human primary macrophages were treated with 1 mM VLDL-sized emulsion particles for 24 hours. The bar graphs were plotted as mean ± SD. Asterisk indicates significantly different in the marked comparisons according to Student t test. *p < 0.05, **p < 0.01. (The raw data can be found in “S1 Raw Data”.) (TIF) [file pbio.3001516.s005.tif]

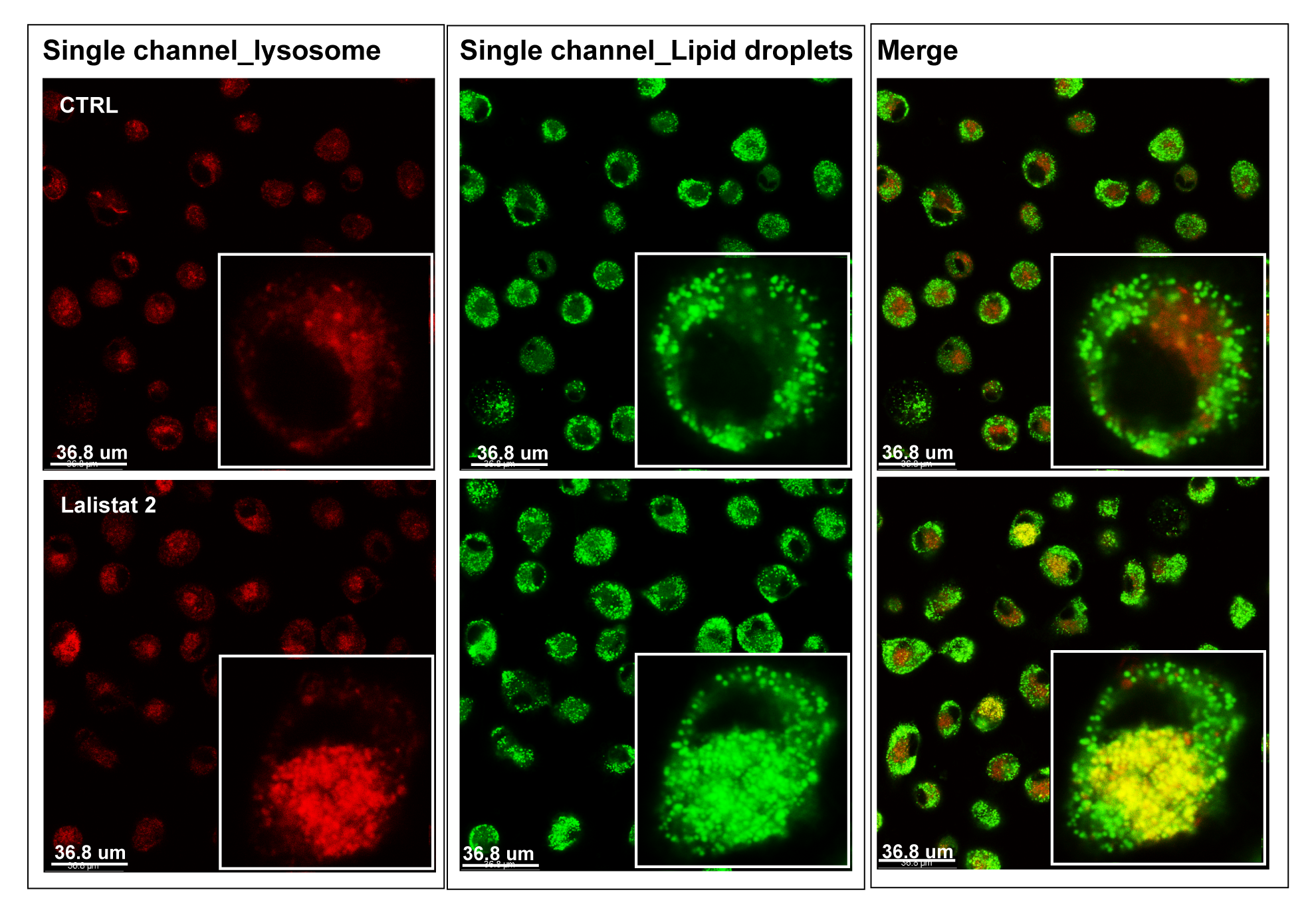

Supplement: S2 Fig — The figure illustrates single channel images of co-staining of lysosome (red) and neutral lipids (BODIPY 493/503, green) in human macrophages in the presence or absence of 30 μM Laslistat 2 (n = 6). Cells were treated with 0.5 mM VLDL-sized emulsion particles for 24 hours. Before imaging, cells were washed twice with PBS and cultured in fresh medium for 24 hours. (TIF) [file pbio.3001516.s006.tif]

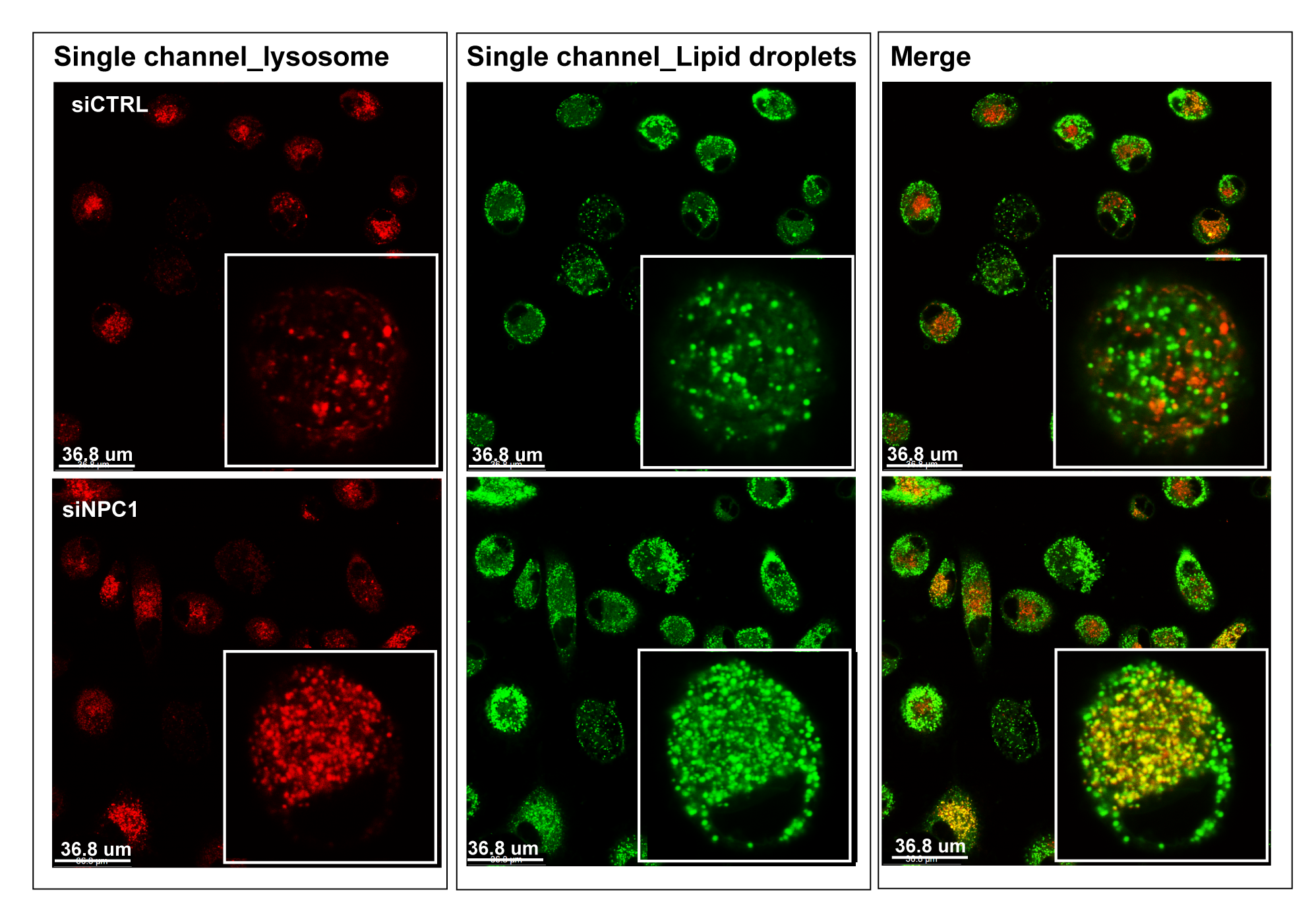

Supplement: S3 Fig — The figure illustrates single channel images of co-staining of lysosome (red) and neutral lipids (BODIPY 493/503, green) in human macrophages treated with siCTRL or siNPC1 for 72 hours, followed by treatment with 0.5 mM VLDL-sized emulsion particles for 24 hours (n = 6). Before imaging, cells were washed twice with PBS and cultured in fresh medium for 24 hours. (TIF) [file pbio.3001516.s007.tif]

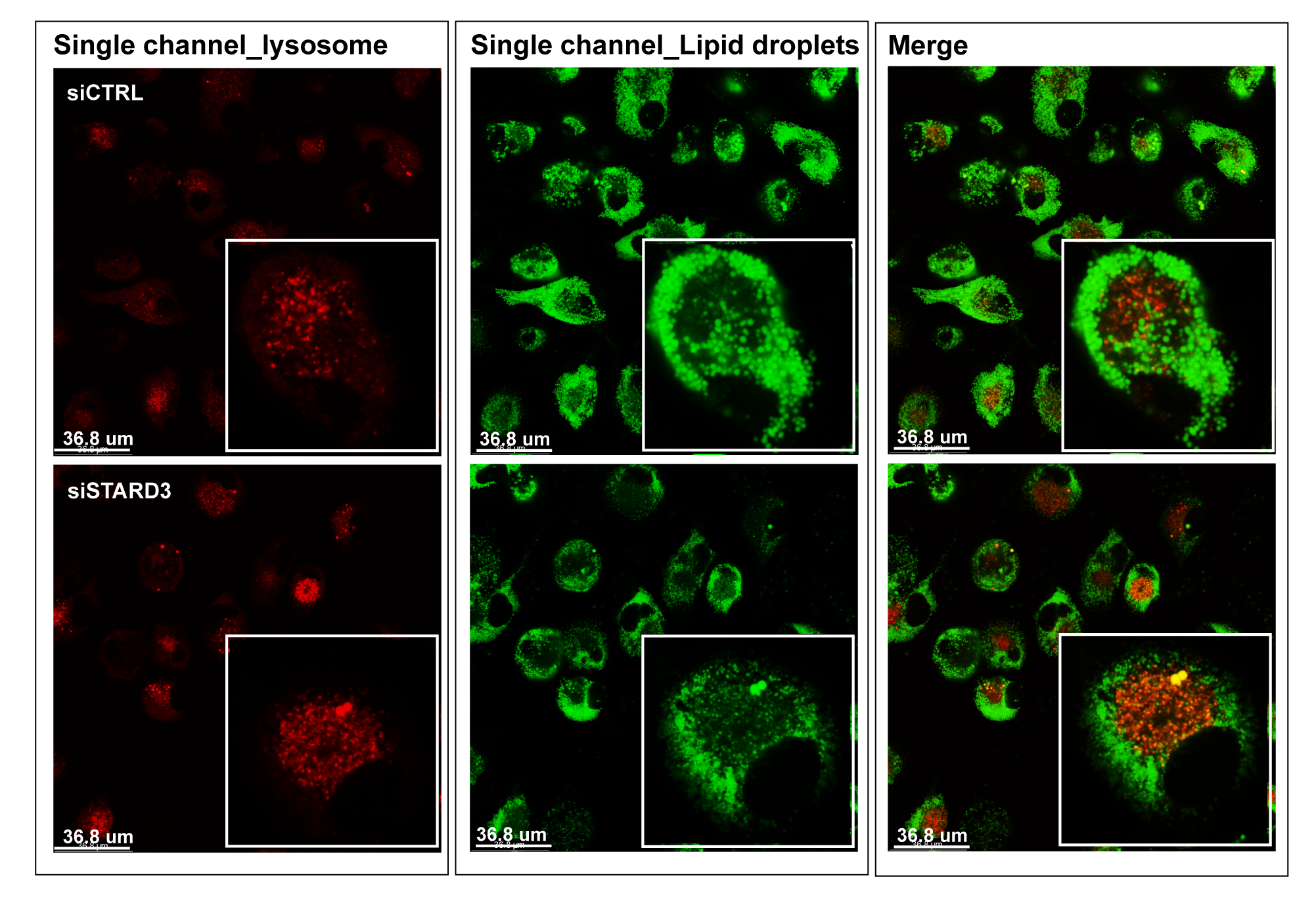

Supplement: S4 Fig — The figure presents single channel images for co-staining of lysosomes (red) and neutral lipids (BODIPY 493/503, green) in human macrophages treated with siCTRL or siSTARD3 for 48 hours and followed by treatment with 0.5 mM VLDL-sized emulsion particles for 24 hours (n = 6). Before imaging, cells were washed twice with PBS and cultured in fresh medium for 24 hours. (TIF) [file pbio.3001516.s008.tif]

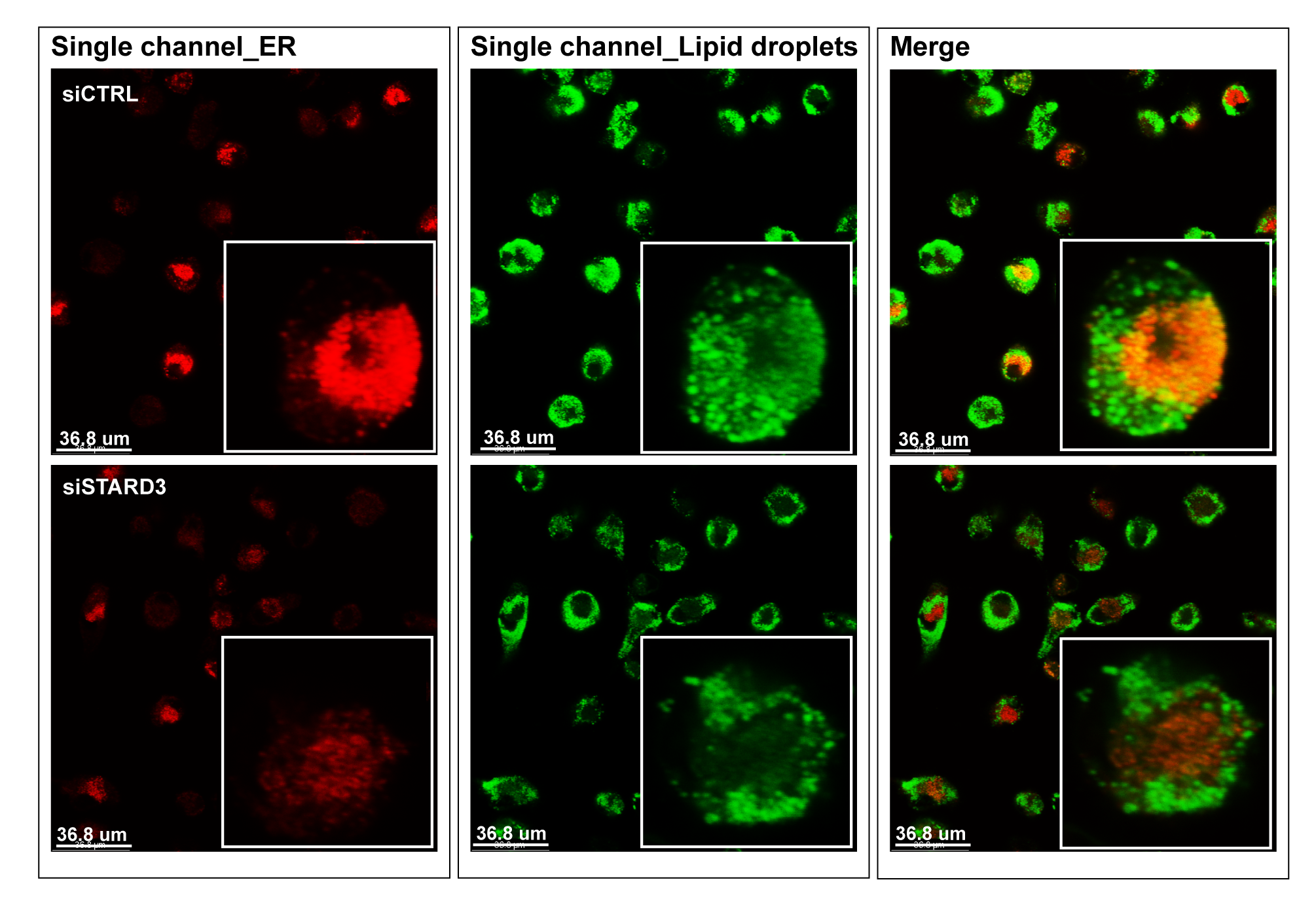

Supplement: S5 Fig — The figure presents single channel images for co-staining of ER (red) and neutral lipids (BODIPY 493/503, green) in human macrophages treated with siCTRL or siSTARD3 for 48 hours followed by treatment with 0.5 mM VLDL-sized emulsion particles for 24 hours (n = 6). (TIF) [file pbio.3001516.s009.tif]

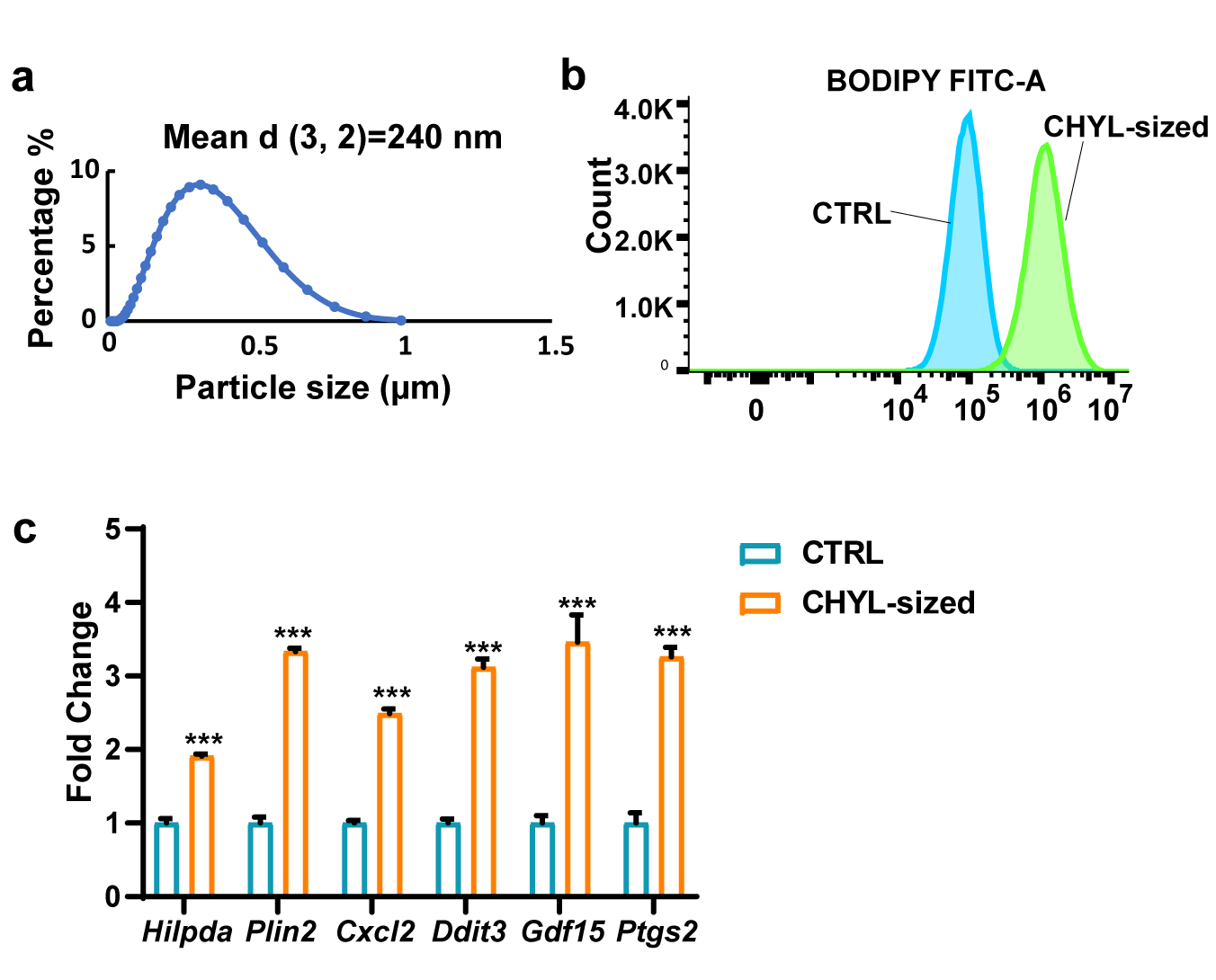

Supplement: S6 Fig — (a) The particle size distribution of CHYL-sized emulsion particles as determined by mastersizer 3000. (b) Mean fluorescence intensity (FITC-A) measured by flow cytometry of mouse RAW 264.7 macrophages treated with 1 mM CHYL-sized emulsion particles for 6 hours (n = 3). (c) mRNA expression of lipotoxic marker genes in RAW 264.7 macrophages. Bar graphs were plotted as mean ± SD. Statistical significance was analysed using 2-way ANOVA; *p < 0.05, **p < 0.01, ***p < 0.001, ***p < 0.0001. (The FACS data are available under repository ID FR-FCM-Z5K3. The raw data of bar graphs can be found in “S1 Raw Data”.) (TIF) [file pbio.3001516.s010.tif]

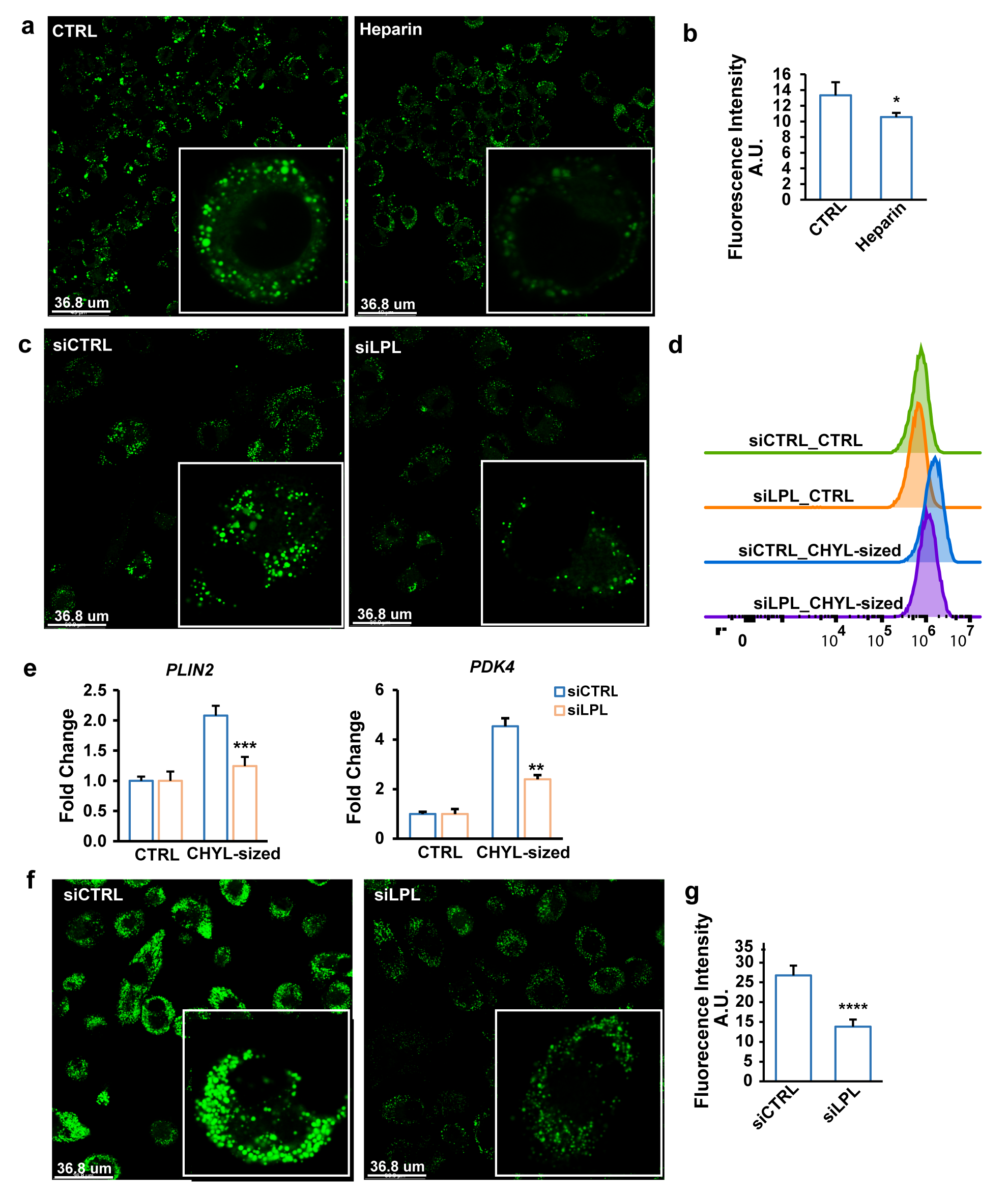

Supplement: S7 Fig — (a) BODIPY 493/503 staining of intracellular neutral lipids in RAW 264.7 macrophages treated with 1 mM CHYL-sized emulsion particles for 6 hours in the presence or absence of 50 UI/ml human heparin (n = 6). (b) Quantification of the fluorescence images by ImageJ (n = 4). (c) BODIPY 493/503 staining of intracellular neutral lipids in human macrophages treated with siCTRL or siLPL for 48 hours followed by treatment with 0.5 mM CHYL-sized emulsion particles for 6 hours (n = 6). (d) Mean fluorescence intensity quantified by flow cytometry (n = 3). (e) mRNA expression of selected lipid-sensitive genes. (f) BODIPY 493/503 staining of intracellular neutral lipids in human macrophages treated with siCTRL or siLPL for 48 hours followed by treatment with 0.5 mM human plasma isolated CHYL for 6 hours (n = 6). (g) Mean fluorescence intensity quantified by Image J (n ≥ 4). The bar graphs were plotted as mean ± SD. Asterisk indicates significantly different from control according to Student t test. *p < 0.05, **p < 0.01, ***p < 0.001. (The FACS data are available under repository ID FR-FCM-Z5KY. The raw data of bar graphs can be found in “S1 Raw Data”.) (TIF) [file pbio.3001516.s011.tif]

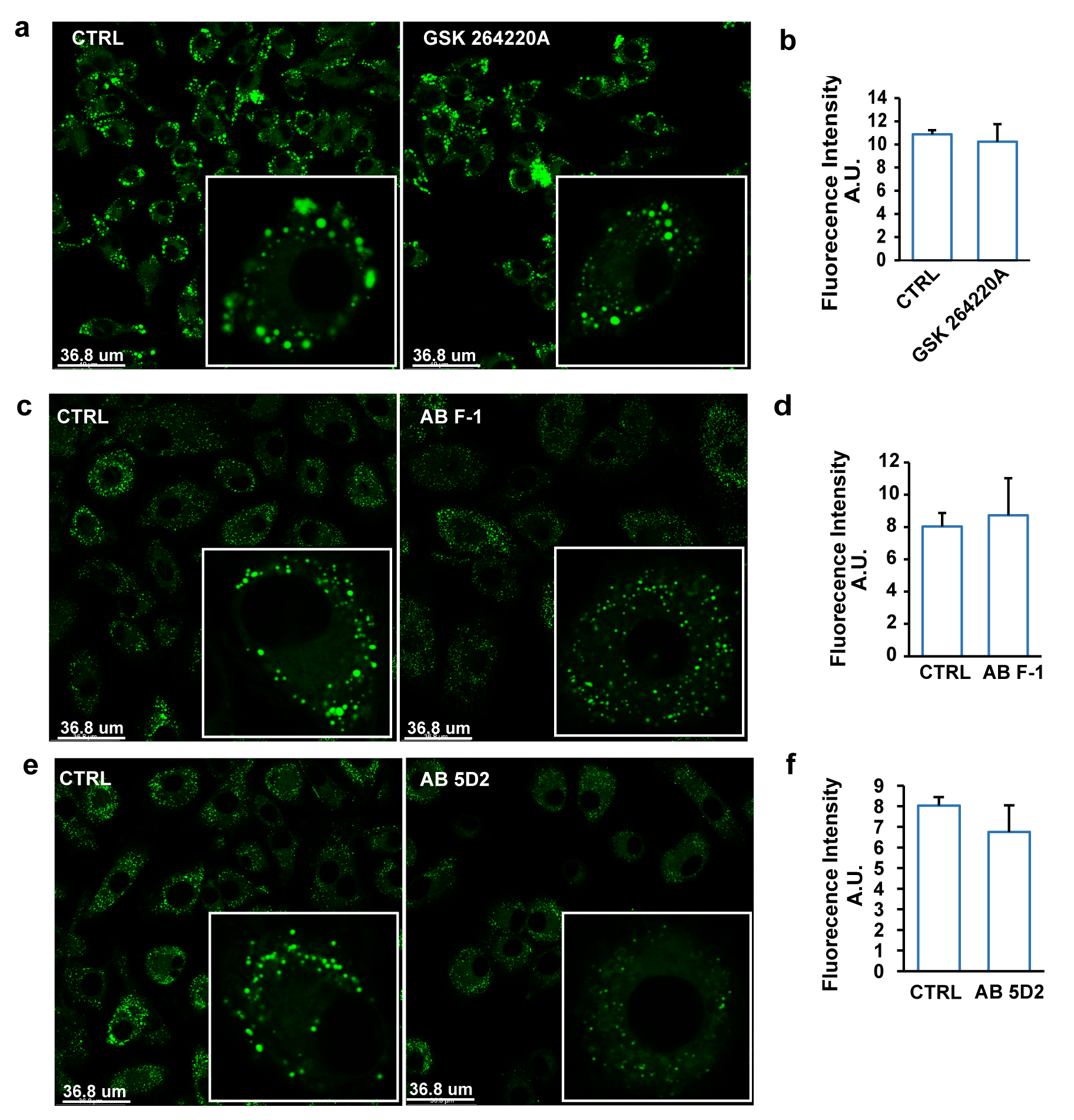

Supplement: S8 Fig — (a) BODIPY 493/503 staining of RAW 264.7 macrophages treated with 1 mM CHYL-sized emulsion particles for 6 hours in the presence or absence of 0.2 μM of the catalytic LPL inhibitor GSK264220 (n = 6). (b) Mean fluorescence intensity quantified by Image J (n ≥ 4). (c) BODIPY 493/503 staining of human primary macrophages treated with 0.5 mM CHYL-sized emulsion particles for 6 hours in the presence or absence of antibody F1 targeting the N-terminal portion of LPL (2 μg/ml) (n = 6). (d) Mean fluorescence intensity quantified by Image J (n ≥ 4). (e) BODIPY 493/503 staining of human primary macrophages treated with 0.5 mM CHYL-sized emulsion particles for 6 hours in the presence or absence of antibody 5D2 targeting the C-terminal portion of LPL (2 μg/ml) (n = 6). (f) Mean fluorescence intensity quantified by Image J (n ≥ 4). The bar graphs were plotted as mean ± SD. Asterisk indicates significantly different from control according to Student t test. ****p < 0.0001. (The raw data of bar graphs can be found in “S1 Raw Data”.) (TIF) [file pbio.3001516.s012.tif]

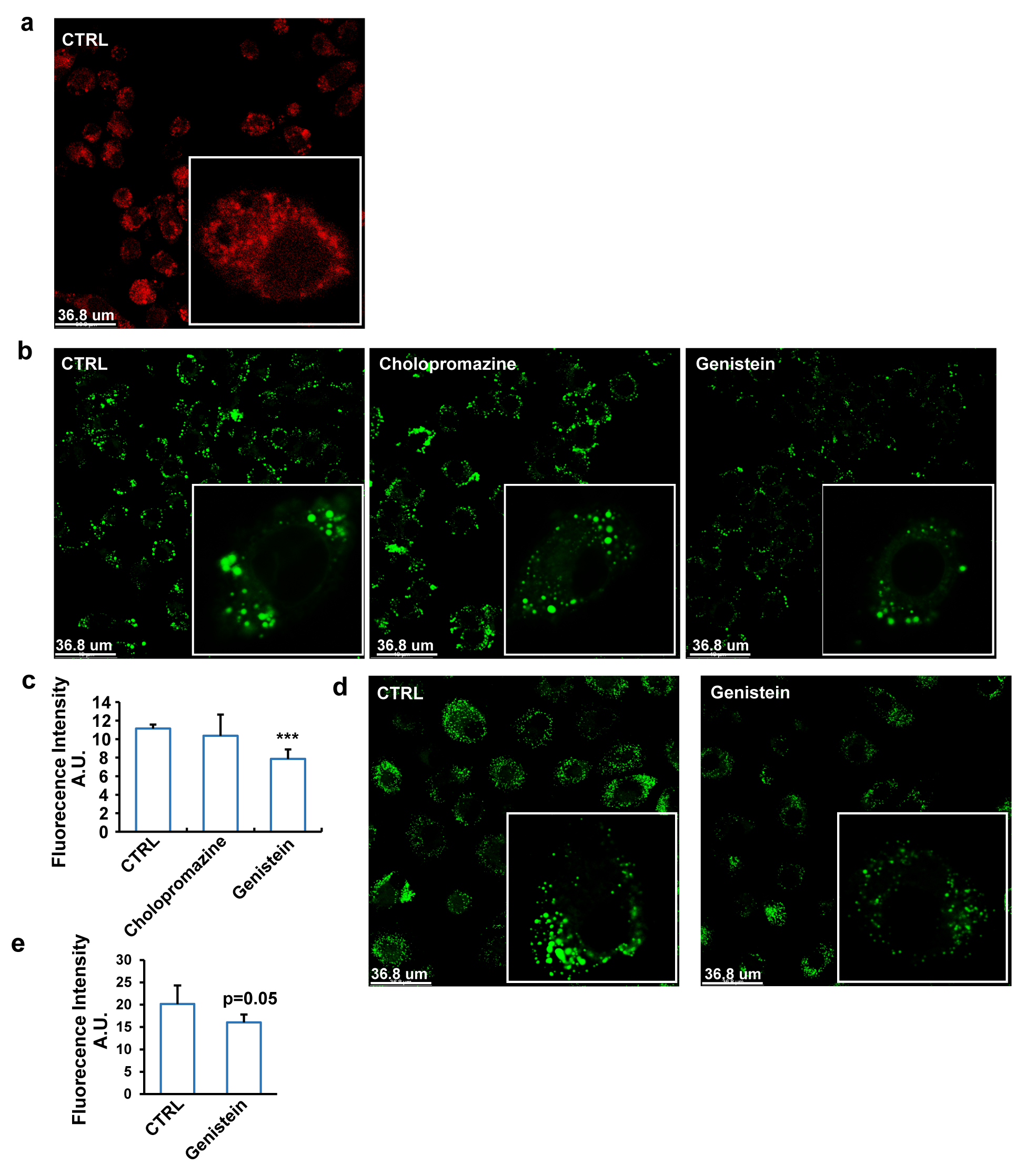

Supplement: S9 Fig — (a) Early endosome staining of RAW 264.7 macrophages treated with 1 mM of CHYL-sized emulsion particles for 6 hours (n = 6). (b) BODIPY 493/503 staining of RAW 264.7 macrophages treated with 1 mM CHYL-sized emulsion particles for 6 hours in the presence or absence of 10 μg/ml chlorpromazine or 200 μM genistein (n = 6). (c) Mean fluorescence intensity quantified by Image J (n = 6). (d) BODIPY 493/503 staining of human primary macrophages treated with 0.5 mM CHYL-sized emulsion particles for 6 hours in the presence or absence of 200 μM genistein (n = 6). (e) Mean fluorescence intensity quantified by Image J (n = 3). The bar graphs were plotted as mean ± SD. ***p < 0.001. (The raw data of bar graphs can be found in “S1 Raw Data”.) (TIF) [file pbio.3001516.s013.tif]

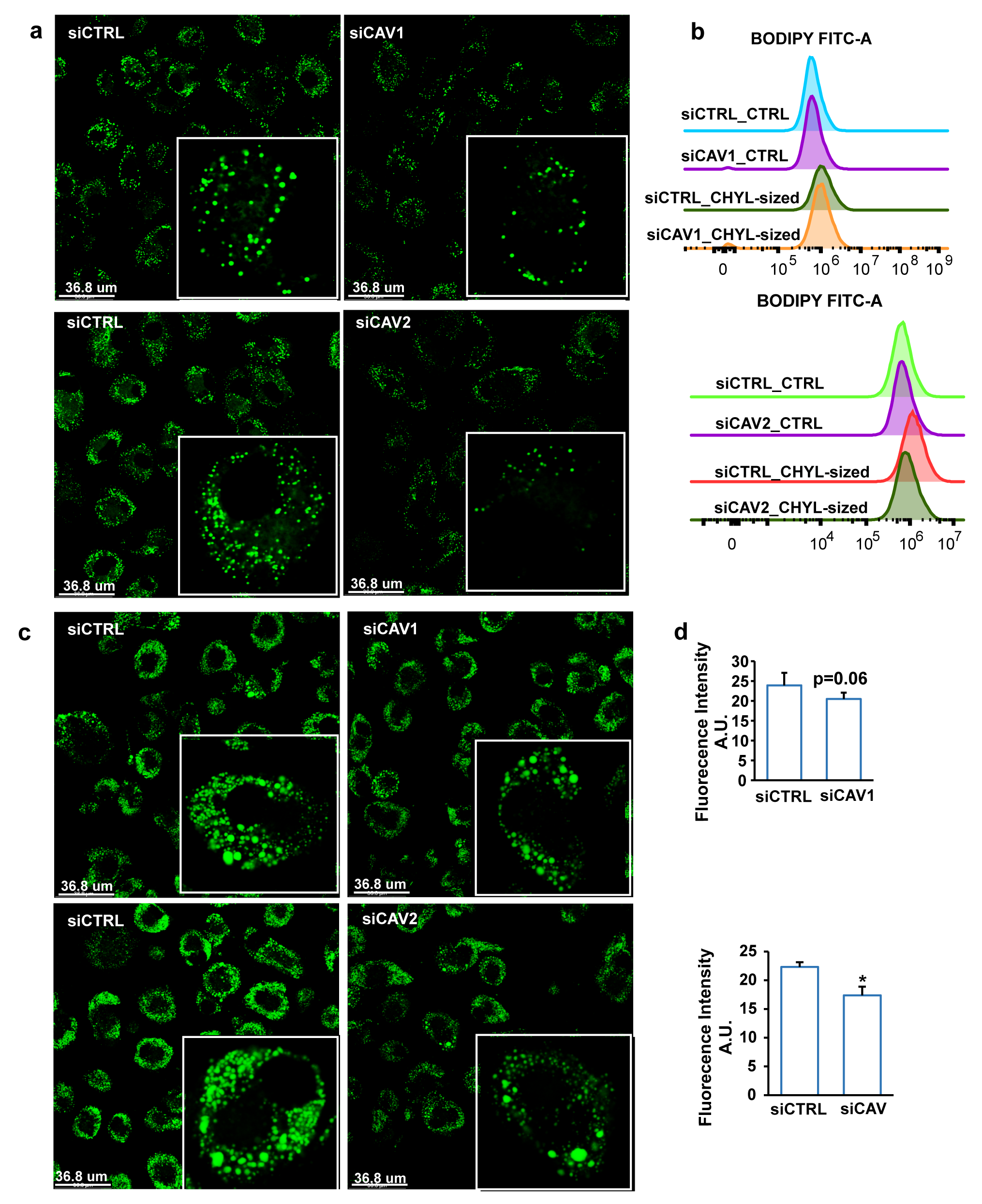

Supplement: S10 Fig — (a) BODIPY 493/503 staining of human macrophages treated with siCTRL, siCAV1, or siCAV2 for 48 hours followed by treatment with 0.5 mM CHYL-sized emulsion particles for 6 hours (n = 6). (b) Mean fluorescence intensity quantified by flow cytometry (n = 3). (c) BODIPY 493/503 staining of human macrophages treated with siCTRL, siCAV1, or siCAV2 for 48 hours followed by treatment with 0.5 mM human plasma-isolated CHYL for 6 hours (n = 6). (d) Mean fluorescence intensity quantified by Image J (n = 4). The bar graphs were plotted as mean ± SD. Asterisk indicates significantly different from control according to Student t test. *p < 0.05. (The FACS data are available under repository FR-FCM-Z5JV and FR-FCM-Z5KZ. The raw data of bar graphs can be found in “S1 Raw Data”.) (TIF) [file pbio.3001516.s014.tif]

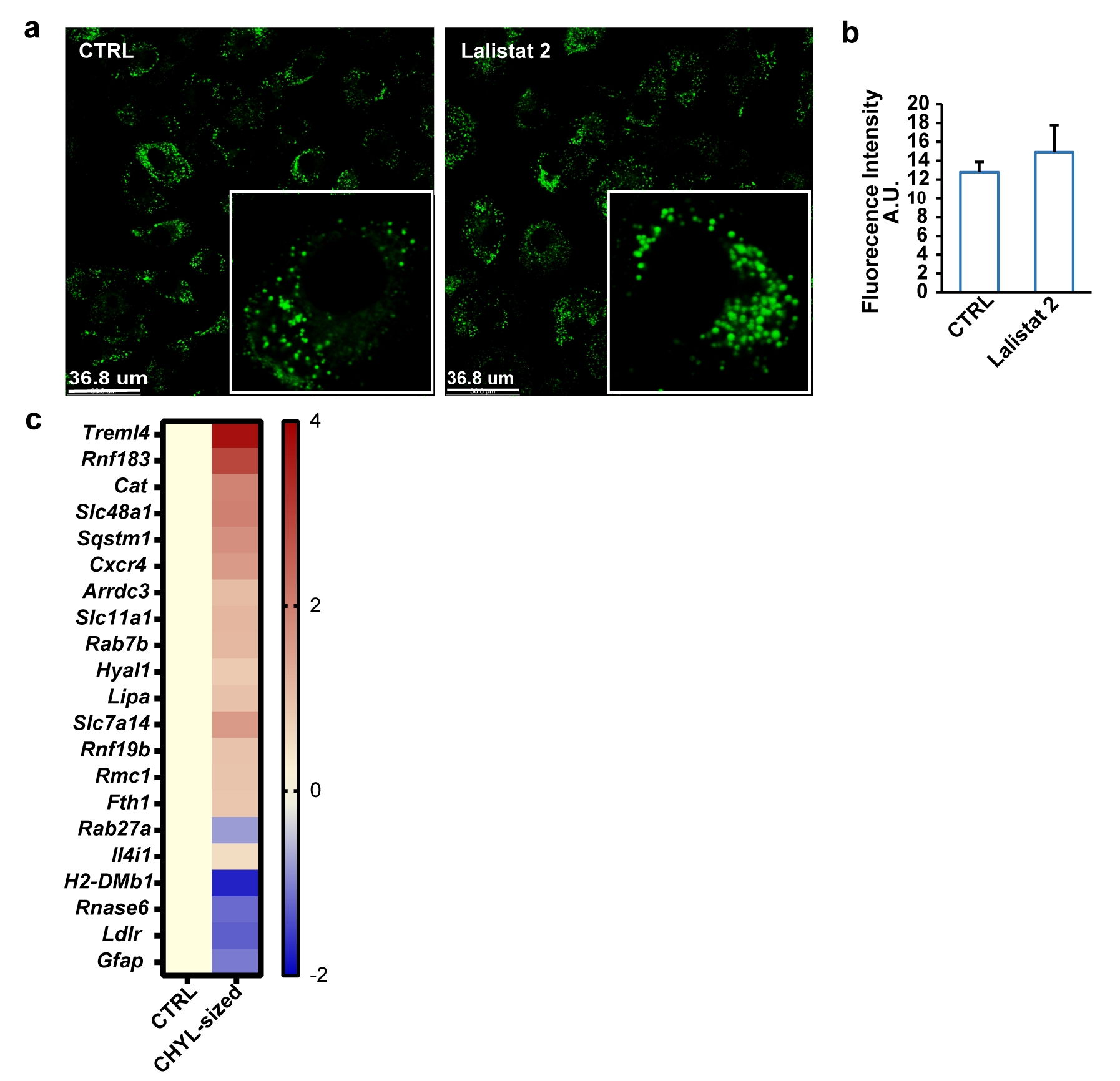

Supplement: S11 Fig — (a) BODIPY 493/503 staining of human macrophages treated with 0.5 mM CHYL-sized emulsion particles for 6 hours in the presence or absence of 30 μM Lalistat 2 (n = 6). (b) Mean fluorescence intensity quantified by Image J (n = 6). (c) Heatmaps showing changes in the expression of genes involved in lysosome activity in RAW 264.7 macrophages treated with 0.5 mM CHYL-sized emulsion particles for 6 hours (p < 0.01, SLR > 1). Scale bar depicts SLR. (The raw data of RNA-sequencing are available under accession number GSE203250. Other data can be found in “S1 Raw Data”.) (TIF) [file pbio.3001516.s015.tif]
