# Supplementary material for: Macrophages take up VLDL-sized emulsion particles through caveolae-mediated endocytosis and excrete part of the internalized triglycerides as fatty acids
Source: PLoS Biol. 2022 Aug 26;20(8):e3001516. doi: 10.1371/journal.pbio.3001516 (PMC9455861; doi:10.1371/journal.pbio.3001516)
Supplement: Attachment — Submitted filename: PBIOLOGY-D-21-03170R2 Rebuttal.docx [file pbio.3001516.s016.docx]

Dear Editor,

Please find enclosed our revised manuscript PBIOLOGY-D-21-03170R1 entitled “Caveolae mediated endocytosis of VLDL particles in macrophages requires NPC1 and STARD3 for further lysosomal processing”. We thank the reviewers for their positive and constructive comments and suggestions, which helped us to greatly improve our manuscript.

In response to the comments from reviewers, we performed the following additional experiments:

1) We co-silenced CAV1 and CAV2 in human primary macrophages and imaged Bodipy 493/503-stained lipid droplets using confocal microscope. The results show that compared with single silencing, the co-silencing further lowers the uptake of the emulsion lipid particles by macrophages, although the difference between co-silencing and CAV2 silencing is minor (Figure 5).
2) We treated primary human GM-CSF with artificial VLDL-sized emulsion particles while silencing LPL, CAV1, or CAV2, and performed RT-qPCR to measure several cytokine/chemokine-related genes (Suppl. Figure 1)
3) We treated human primary GM-CSF with VLDL-sized emulsion particles and LAL inhibitor to further validate that the free fatty acids detected in the medium are lysosomal-derived. LAL inhibition significantly reduced free fatty acid levels in medium of cells, further supporting the notion that macrophages can secrete lysosomal hydrolyzed fatty acids (Figure 6e).
4) We redid the LPL Western blot of LPL-silenced primary macrophages samples to produce higher quality blots (Figure 2d).

As requested by one of the reviewers, we also changed the description of the emulsion particles to VLDL-sized emulsion particles (VLDLsep). In addition, we made other textual changes to the manuscript in accordance with the comments of the reviewers. As required, we also added the target sequences of siRNA used and the single channel images of co-stainings.

Please find below a point-by-point response to the reviewer’s comments. We hope that our revised manuscript now meets the high-quality standards to publish in PLoS Biology. Thank you for taking our manuscript into consideration.

Sincerely,

Sander Kersten

**Reviewers’ comments**

Rev. 1: Alison Kohan – note that this reviewer has signed her review Overall, the hypotheses tested in this manuscript are impactful and suggest serious reconsideration of the triglyceride metabolic pathways in macrophages and how these may be functioning in diseases like atherosclerosis. The idea that macrophages have a dedicated FFA secretion process that is part of their normal uptake/metabolism of triglyceride-rich lipoproteins is intriguing. The authors use VLDL-sized casein-emulsified particles (which they call "VLDL-mimic" particles) to study the uptake and intracellular metabolism of neutral lipids in both RAW and human macrophages. In a few instances, the authors also confirm their finding using human VLDL. The authors use chemical and siRNA inhibition to block key steps in endocytosis (LPL activity and caveolin), transport to and hydrolysis in the lysosome (LAL, NPC1), and movement between organelles (using stains for lysosome, nucleus, neutral lipid) and secretion into the media (NEFA assays). Overall, the authors conclude that emulsion particle-derived lipids can be endocytosed, transported to the lysosome, and when normal metabolism to/from the lysosome is blocked (via NPC1 and STARD3), the FFA can't be secreted nor travel to the ER. The authors have developed an interesting hypothesis and I agree with their assessment that the mechanisms governing metabolism of lipoprotein TAG by immune cells is underdeveloped. However, I have some criticisms of the way the quite of experiments were carried out.

(1) Other than sharing a similar size, the VLDL-sized emulsion particles (coated with casein) do not share significant similarities to physiological VLDL. The emulsion particles have no cholesterol, no apolipoproteins, and the phospholipid source is unique from VLDL. These particles are more like milk than VLDL. The Discussion section acknowledges the drawbacks of using VLDL-mimic particles, and the authors have fully and clearly described these drawbacks. They also used human VLDL to duplicate their finding that LPL is required. This lends some confidence. However, the authors haven't duplicated other key findings using this approach. Considering this issue, I would caution against calling them VLDL-mimics. My opinion would be to remove the term "VLDL" or "VLDL-mimic", and instead adjust the title, figures, and annotations to refer to "emulsion particles" or rather than VLDL.

**Our response:** We thank the reviewer for the valuable suggestion. We agree that we cannot be certain that our emulsion particles fully mimic the behavior of VLDL in all our assays. As an alternative, we suggest the term VLDL-sized emulsion particles, which we abbreviate as VLDLsep. In the revised manuscript, we have replaced VLDL-mimic by VLDLsep, including in the title.

(2) Some details about methods and experiments appear to be missing. In the Methods section there is not enough information about how the emulsion particles were made, and why casein was chosen as the emulsifier. How was the lipid dried, solubilized, vortexed, etc.? There could also be more information about the co-stains used for neutral lipids versus lysosomes. Is there evidence for the specificity of the lysosomal stains (versus cross-staining of ER)? Finally, I can't find the experimental number (n=? in each figure legend). Assuming the n is reasonable, the statistics seem reasonably done and interpreted. Figure 8d is referred to in the text but isn't described in its figure legend. Critically, this figure purports to show transit from the lysosome to ER, but it's unclear whether the stain is still the lysosomal stain or an ER-specific stain.

**Our response:** We thank the reviewer for raising these points. Plasma lipoproteins are protein-lipid emulsions. While most studies use phospholipids as emulsifier, we used a neutral protein source, for which we chose casein. In pilot studies, whey protein was similarly effective in supporting formation of the emulsion particles. Please note that the lipid emulsions in milk do not contain either casein or whey. Accordingly, our emulsion particles do not resemble the lipid emulsion particles in milk. During preparation of the emulsion particles, we did not dry the lipids. Instead, we pre-emulsify the sunflower oil in caseinate-PBS solution using Ultra Turrax, followed by preparation of the actual emulsion particles using a microfluidizer. In the revised manuscript, further details on the manufacturing process of the emulsion particles have been added (page 16).

The LysoTracker probes consist of a fluorophore linked to a weak base that is only partially protonated at neutral pH. This allows LysoTracker probes to freely permeate cell membranes enabling them to label live cells. LysoTracker probes are highly selective for acidic organelles. Considering the lysosome is the only organelle that is acidic, it is reasonable to consider that LysoTracker specifically stains lysosomes. This information has been added to the methods section (page 21).

In the revised manuscript, we have adding the number of replicates (n) in the figure legends.

(3) In some cases, the data could be interpreted in a variety of ways. For example, when NPC1 is inhibited and NEFA rises in the media, this doesn't necessarily mean that the FFA is being actively secreted from intracellular pools within the macrophage. It could mean that there is a change in the rate of FFA generation in the media (from VLDL precursor), or that FFAs (derived from VLDL in the media) are being retained in the media rather than being endocytosed. It would be helpful for this to be discussed. Similarly, because the authors use a neutral lipid stain it is hard to dissect differences between the accumulation of triglycerides and cholesterol, and in some cases the authors conclude that the stains show the movement of FFA between cellular locations. Without specific radio- or fluorescent labels, or more information about the variety of stains used, I'm unsure about how to interpret this. They do bring this issue up in the Discussion, but this concern should probably be a part of the Results as well.

**Our response:** We thank the reviewer for raising this very relevant point. In order to exclude that the free fatty acids present in the medium are generated in the medium from the lipid emulsion particles, we washed the cells extensively prior to incubation with medium devoid of lipid emulsion particles. Using this protocol, it is highly plausible that the free fatty acids detected in the cell culture medium have an intracellular origin. As an additional validation, macrophages incubated with VLDLsep were also treated with the LAL inhibitor Lalistat 2 after changing the medium to VLDLsep-free medium. Consistent with the other data, we found that the increase in free fatty acids in the culture medium could be abolished by LAL inhibition, supporting the notion that the fatty acids in the medium are derived from the lysosome. These new data are presented in Figure 6e and add to a growing body of evidence that non only adipocytes but also macrophages, hepatocytes, and endothelial cells may be able to release fatty acids (PMID: 6508637, 28119423, 32070194).

We have thought very carefully about the use of fluorescently-labelled fatty acids but unfortunately, they are only available as fatty acids and not as part of triglycerides. Concerning the use of radiolabelled triglyceride emulsion particles, their use suffers from the same potential complication highlighted by the reviewer: that the fatty acids are generated extracellularly and not intracellularly. Accordingly, the specific steps in our protocol (extensive washing, LAL inhibition) are crucial to be able to conclude that the fatty acids in the medium are derived from intracellular lipolysis.

In summary, despite these concerns, the findings in this manuscript add to our knowledge about extracellular triglyceride in regulating intracellular metabolism in macrophages. After reading the manuscript I kept thinking about how it might impact my own and others' work - so there is certainly potential impact. In my opinion, this manuscript (with some revisions) should be published.

Rev. 2:

I have read with interest the manuscript of Lei Deng, et al.

The manuscript shows that uptake of VLDL by the macrophage is mediated by the C terminal of LPL via caveolar-mediated endocytosis. Interestingly, NPC1 was found to promote the extracellular efflux of fatty acids from lysosomes, while Stard3 is involved in the transfer of lysosomal fatty acids to the ER for subsequent storage as triglycerides. I feel that authors' manuscript is well performed and contains interesting data from a basic research perspective. However, in my point of view, several limitations will negatively affect the study and some claims are overstated based on their evidence.

The main issue about this manuscript:

1. Much of the cited literature that is used to make the case for their hypothesis is very old and actually refers to macrophage uptake the VLDL and LPL serve as the bridge for that.

**Our response:** We thank the reviewer for this comment. It is true that we built our manuscript on findings that were made a long time ago about how macrophages process TG-rich lipoproteins. Considering the growing recognition of the role of TG-rich lipoproteins in atherosclerosis (PMID: 34472586), we decide to reactivate this question (page 4). Part of our manuscript confirms these findings using modern-day techniques. The other part adds to the findings and delivers important new mechanistic insights into this process.

2. To my recollections, several papers mentioned that 5D2 binds to human LPL but fails to bind to mouse LPL. Because RAW 264.7 cells were derived from BALB/c mice and only express mouse LPL. Therefore, it will be confused to interpret the data (Figure 3e) that 5D2, definitely failed to block the C-terminal of mouse LPL but impaired the vLPLm uptake on mouse macrophages. It cannot be the strong support for the main idea. Thus, the statement that C-terminal region of mouse LPL is required for macrophage uptake of vLDLm-TG is not supported by the data

**Our response:** The reviewer is making an excellent point here. In the legend of Figure 3e, we mistakenly state RAW macrophages while it should be human primary macrophages. We very much apologize for this mistake. It is true that 5D2 is specific for human LDL but since we used human cells, there is no concern here. Thank you for bringing this to our attention.

3. Detecting the medium FFA and genes expression levels cannot be the strong support for that NPC1 was found to promote the extracellular efflux of fatty acids from lysosomes, while Stard3 is involved in the transfer of lysosomal fatty acids to the ER for subsequent storage as triglycerides.

**Our response:** We thank the reviewer for this valuable comment. The experimental basis for the notion that NPC1 promotes the extracellular efflux of fatty acids is that silencing and inhibition of NPC1 attenuates the VLDLsep-induced increase in free fatty acid levels in the medium. The data indicate that NPC1 somehow promotes the release of fatty acids by macrophages. We agree that these data do not allow us to draw firm conclusions about whether NPC1 directly or indirectly supports this process. Accordingly, in the revised manuscript, we have changed the wording to accommodate this concern. For example, in the results it is written “Although these data do not necessarily indicate that NPC1 directly mediates free fatty acids efflux from lysosome, they do suggest that NPC1 is involved in promoting the extracellular release of fatty acids from macrophages.“ In the discussion we write that “we found that macrophages loaded with VLDLsep release fatty acids into the medium and additionally observed that this process was impaired by inactivation of LAL and NPC1. At this stage, it is unclear if extracellular fatty acid efflux is dependent on the fusion of the lysosomes to the plasma membrane or requires fatty acid transport through the cytoplasm. Since NPC1 was specifically dismissed as fatty acid transporter [55], the decreased fatty acid release upon NPC1 inactivation is probably secondary to impaired lysosomal lipolysis due to lysosomal accumulation of cholesterol, rather than reflecting a direct role of NPC1 as fatty acid transporter. Previously, it was suggested that lipid-laden macrophages may release fatty acids via the action of a nonlysosomal (presumably cytoplasmic) neutral triglyceride lipase. Based on our data, we cannot exclude that the increase in fatty acid release upon NPC1 silencing was dependent on TG storage in lipid droplets and ATGL-mediated lipolysis prior to the release of the fatty acids into the medium.”

The experimental basis for the notion that Stard3 is involved in the transfer of lysosomal fatty acids to the ER is that Stard3 silencing increased the accumulation of neutral lipids in lysosomes, while reducing lipids in the ER. Unlike silencing of NPC1, silencing of Stard3 did not increase intracellular lipid accumulation nor did it influence efflux of fatty acids into the medium. We agree that we strictly cannot conclude that the lipids in the ER are directly derived from the lysosome. It is also possible that the lysosome-derived free fatty acids have been trafficked to some other cellular compartment, which indirectly increases the lipid storage in ER. However, given the established role of Stard3 in transporting cholesterol directly from the lysosome to the ER, our data are most consistent with a similar role of Stard3 in fatty acid transport. We have changed the text to accommodate this concern (page 10).

4. The authors treated the RAW264.7 macrophages with 50 UI/ml of heparin for 2 hours, LPL protein levels decreased by ~20%. Several groups demonstrated that LPL can be internalized into cells, it is highly recommended to perform the immunofluorescent staining or live cell fluorescence binding experiment rather than Western blot, if you want to test the release effect of heparin on LPL.

**Our response:** We appreciate this valuable suggestion. As indicate by the reviewer, heparin is able to activate the internalization/degradation and release of LPL (PMID: 8089154). In our experiment, LPL protein was measured in the whole lysate. Hence, regardless of the mechanism, we can conclude that heparin treatment leads to less LPL being available to supporting lipid uptake, which is what we wanted to achieve. We have altered the text and specifically mention the two possible mechanisms by which heparin may reduce surface LPL abundance (page 6).

5. The authors explained that "the LPL remaining after heparin treatment likely represents the functionally inactive intracellular LPL pool". Authors should present their data to support this idea. Previous studies showed that high levels of heparin (500U/ml) fully dissociate LPL from cell surface. We suspected that the authors could only release some proportion of LPL, the remaining LPL might be still functional.

**Our response:** We agree with the reviewer that we cannot claim that the LPL remaining after heparin treatment represents the functionally inactive intracellular LPL pool. We also agree that the remaining LPL could still be functional, which may explain why lipid uptake wasn’t completely abolished. We have removed the sentence about inactive LPL pool.

6. In Figure2D, the LPL bands were smeared. This data is even less convincing.

**Our response:** We agree that the quality of the LPL Western blot was not optimal. We have rerun the samples and the new Western blot looks more clear. It has been inserted in Figure 2D.

7. Silencing of both Caveolin 1 and 2 will impair uptake of VLDL further, compared with single siRNA treatment?

**Our response:** The reviewer raises a very relevant question. We performed co-silencing experiments of CAV1 and CAV2 and observed a very clear reduction in Bodipy staining, indicating a marked decrease in the uptake of the lipid emulsion particles (Figure 5c). Since CAV2 silencing already reduced Bodipy staining a lot, no noticeable further decrease was observed upon co-silencing of CAV and CAV2.

8. Did BODIPY signals co-localize with cav-1 or cav-2 on macrophages loaded with vLDLm?

**Our response:** We thank the reviewer for this question. Interestingly, previous studies have found that CAV1 is enriched in lipid-droplet fractions of endothelial cells and adipocytes (PMID: 16643278, 28119423). It can be hypothesized that in macrophages, part of the CAV1 is similarly associated with lipid droplets, which might imply that the role of caveolins in lipid processing in macrophages may go beyond endocytosis. The above text has been inserted in the discussion (page 13). Unfortunately, we were not successful in immunofluorescent staining of Caveolins in macrophages.

9. The author should also present single-channel images of BODIPY and lysosome marker staining on Figure 6D and Figure 7E. Otherwise, it will be difficult to interpret those data. In Figure 6d left panel, could authors observe the colocalization of BODIPY and lysosome marker?

**Our response:** We appreciate this suggestion by the reviewer. Because of space limitation, we had opted not to show the single channel images but we fully agree with the reviewer that it would be worthwhile for the reader to add these images. In the revised manuscript, the single-channel images of figure 6d, 7e, 8c, and 8d are included as supplemental figures S2-S5. Minimal overlap in lipid- and lysosomal staining was observed in the absence of LAL inhibition (as shown in Figure 6d & Figure S2)

10. In Figure 7E, right panel, it showed heterogenetic co-localization on macrophages treated with siNPC1 followed by VLDL treatment? Please explain that. We recommended to knockout those genes via Crispr. RNA interference and inhibitors did not become clear to me since low transfection efficacy and low specificity might negatively affect the results.

**Our response:** We thank the reviewer for these relevant questions. We suspect that the heterogenetic co-localization is due to heterogeneity of siRNA-based silencing (presumable caused by differences in uptake of siRNA) and heterogeneity in the uptake and processing of lipids.

We agree that in principle CRISPR-based gene editing can be more effective than siRNA and the use of inhibitors. However, it should be realized that we are working with primary human macrophages, which are notoriously difficult to transfect and for which the effective delivery and expression of the Cas9-gRNA ribonucleoprotein (RNP) complex can be problematic. After considerable optimization, we managed to successfully silence genes in human primary macrophages using siRNA. In the future, though, we are interested in moving to CRISPR-based gene editing if we can manage to overcome the technical hurdles.

11. Please add the scale bar to the immunofluorescence images clearly.

**Our response:** We apologize for omitting the scale bars. This has been corrected in the revised manuscript.

12. In supplementary Figure S1G, the authors demonstrated that vLPLm or CHYLm treatment on macrophages induced the expression levels of ER stress inflammatory response related genes, did you observe the similar trend of cytokine expression (i.e., TNF-a, IL-1b, MCP-1, MCP-1, and MMP-3)? Did LPL siRNA, genistein, siCAV1, siCAV2 or 5D2 treatment rescue the phenotype?

**Our response:** Thank you for raising this question. In response, we measured the expression of a number of cytokines in the VLDLsep-treated macrophages and assessed the impact of LPL, CAV1, and CAV2 silencing. The results for IL6 and MCP1 are shown in Supplemental figure S1 and are described on page 8.

13. The figure citation in the manuscript is in ascending numerical order. The authors should citate the figure S1a first.

**Our response:** We apologize and have adjusted the numbering.

14. Ref 13 and ref 19 are duplicated.

**Our response:** We apologize and have adjusted the reference numbers

15. siRNA sequences targeted for LPL, CAV1, CAV2, NPC1 and STARD3 should be required.

**Our response:** The siRNA sequences have been incorporated in the revised manuscript as Table S1.
