# Supplementary material for: Macrophages take up VLDL-sized emulsion particles through caveolae-mediated endocytosis and excrete part of the internalized triglycerides as fatty acids
Source: PLoS Biol. 2022 Aug 26;20(8):e3001516. doi: 10.1371/journal.pbio.3001516 (PMC9455861; doi:10.1371/journal.pbio.3001516)
Supplement: Attachment — Submitted filename: Response to reviewer.docx [file pbio.3001516.s017.docx]

Dear Reviewer,

We greatly appreciate your comments and suggestions. Please find our point-to-point response to your comments.

Reviewer 2:

1. For the response to the question 1 from review 1, I prefer to refer to "emulsion particles" as reviewer1 suggested rather than "VLDLsep" which may cause readers' misunderstanding. As suggested by reviewer1, the emulsion particles have no cholesterol, no apolipoproteins, and the phospholipid source is unique from VLDL.

Our response: Thank you for the suggestion. We agree that “VLDLsep” may cause misunderstanding among some readers. Accordingly, we do not use ‘VLDLsep’ as the abbreviation for VLDL-sized emulsion particles anymore throughout the text and in all the figures. The abbreviation has been replaced by the full name ‘VLDL-sized emulsion particles’. Only in the figures, because of the limited space, we used VLDL-sized, which is illustrated in the figure legend when it appears for the first time. We believe that referring to VLDL in the name of the artificial particles is justified based on the size and composition of the emulsion particles and the confirmation of our key data with actual VLDL particles. It is also a common practice in the field.

2. For the response to the question 2 from review1, it seems that the author did not response to "It could mean that there is a change in the rate of FFA generation in the media (from VLDL precursor), ".

Our response: It is true that we did not specify “VLDL precursor” in our answer.

However, as we explained, we washed the cells extensively prior to incubation with medium devoid of lipid emulsion particles. Using this protocol, it is highly plausible that the free fatty acids detected in the cell culture medium have an intracellular origin. In the meantime, as an additional validation, macrophages incubated with VLDL-sized emulsion particles were also treated with the LAL inhibitor Lalistat 2 after changing the medium to emulsion particle-free medium. Consistent with the other data, we found that the increase in free fatty acids in the culture medium could be abolished by LAL inhibition, supporting the notion that the fatty acids in the medium are derived from the lysosome. These new data are presented in Figure 6e and add to a growing body of evidence that not only adipocytes but also macrophages, hepatocytes, and endothelial cells may be able to release fatty acids (PMID: 6508637, 28119423, 32070194).

Sincerely,

Sander Kersten, on behalf of all co-authors
